# Insecticide resistance evolution with mixtures and sequences: a model-based explanation

**DOI:** 10.1186/s12936-018-2203-y

**Published:** 2018-02-15

**Authors:** Andy South, Ian M. Hastings

**Affiliations:** Department of Parasitology, Liverpool School of Tropical Medicine, Liverpool, L3 5QA UK

**Keywords:** Insecticide resistance, Public health, Mosquitoes, Vector-borne diseases, Infectious diseases, Malaria, Population genetics

## Abstract

**Background:**

Insecticide resistance threatens effective vector control, especially for mosquitoes and malaria. To manage resistance, recommended insecticide use strategies include mixtures, sequences and rotations. New insecticides are being developed and there is an opportunity to develop use strategies that limit the evolution of further resistance in the short term. A 2013 review of modelling and empirical studies of resistance points to the advantages of mixtures. However, there is limited recent, accessible modelling work addressing the evolution of resistance under different operational strategies. There is an opportunity to improve the level of mechanistic understanding within the operational community of how insecticide resistance can be expected to evolve in response to different strategies. This paper provides a concise, accessible description of a flexible model of the evolution of insecticide resistance. The model is used to develop a mechanistic picture of the evolution of insecticide resistance and how it is likely to respond to potential insecticide use strategies. The aim is to reach an audience unlikely to read a more detailed modelling paper. The model itself, as described here, represents two independent genes coding for resistance to two insecticides. This allows the representation of the use of insecticides in isolation, sequence and mixtures.

**Results:**

The model is used to demonstrate the evolution of resistance under different scenarios and how this fits with intuitive reasoning about selection pressure. Using an insecticide in a mixture, relative to alone, always prompts slower evolution of resistance to that insecticide. However, when resistance to both insecticides is considered, resistance thresholds may be reached later for a sequence relative to a mixture. Increasing the ability of insecticides to kill susceptible mosquitoes (effectiveness), has the most influence on favouring a mixture over a sequence because one highly effective insecticide provides more protection to another in a mixture.

**Conclusions:**

The model offers an accessible description of the process of insecticide resistance evolution and how it is likely to respond to insecticide use. A simple online user-interface allowing further exploration is also provided. These tools can contribute to an improved discussion about operational decisions in insecticide resistance management.

## Background

Insecticide resistance is a problem for malaria control [1–3] other vector borne diseases [4] and agriculture [5]. Malaria alone still results in hundreds of thousands of deaths per year. Recent malaria control efforts have centred on treated bed nets and indoor residual spraying, both reliant on insecticides. Treated nets were recently estimated to contribute 68% and indoor residual spraying 13% to averting more than 500 million falciparum malaria cases between 2000 and 2015 [6]. A recent malaria transmission model [7] predicts that even low pyrethroid resistance is likely to increase malaria incidence in Africa by reducing the performance of bed nets.

The WHO produced a Global Plan for Insecticide Resistance Management in malaria vectors (GPIRM) [1], which includes recommendations on operational strategies for managing resistance including the use of insecticide mixtures when they become available. Efforts are under way to develop new insecticides that will be effective in the light of existing resistance and allow additional options within insecticide resistance management. The Innovative Vector Control Consortium (IVCC) was set up in 2005 to work with industry to develop new vector control tools and particularly new insecticides with new modes of action to address insecticide resistance in disease transmitting mosquitoes [8, 9]. Three new insecticides are now in development [9] and likely to be available after 2020 [2]. It is important that decisions about how best to use the new insecticides, with a clear mindset of delaying the evolution of resistance, are made before insecticides are released [3].

A recent comprehensive review of strategies to avoid resistance evolution across pesticides and drugs [10] concluded that mixtures (combination of molecules) are usually the best resistance management strategy. This was based on both empirical and modelling work. Modelling studies have investigated the evolution of insecticide resistance in insecticide mixtures including in a public health context (e.g. [11–13]), but much of the work was done more than 25 years ago and there remained some confusion about the results [14]. In a recent paper [14], the technical details of a flexible model used to investigate the relative benefits of mixtures and sequences were described. For that paper, thousands of scenarios were run to explore potential outcomes. In contrast, this paper provides an accessible summary of the model and uses selected parameter values to describe mechanistically how the evolution of resistance can be expected to respond. This mechanistic understanding can contribute to the debate on the relative merits of different insecticide strategies and extend existing frameworks [1, 4, 5, 10, 13]. Making the model more accessible, is an attempt to help bridge gaps between academia and policy in insecticide resistance management [15].

This modelling approach focuses on the change in frequency of a single resistance gene for each insecticide. It assumes that genes conferring resistance are already present in the population and that resistance to each insecticide is coded by a single gene. These assumptions are consistent with previous modelling work [10]. In support of the first assumption insecticide resistance has been termed ‘pre-adaptive’ meaning that resistance alleles are present in very low numbers, most likely as a mutation-selection balance, even prior to exposure to novel insecticides [16, 17], as has been documented in blowflies [18]. In support of using a single gene representation of resistance, evidence suggests that whilst polygenic resistance is common, control failures, particularly those due to target site resistance, are mostly due to single major genes [16].

## Methods

**Fig. 1:**
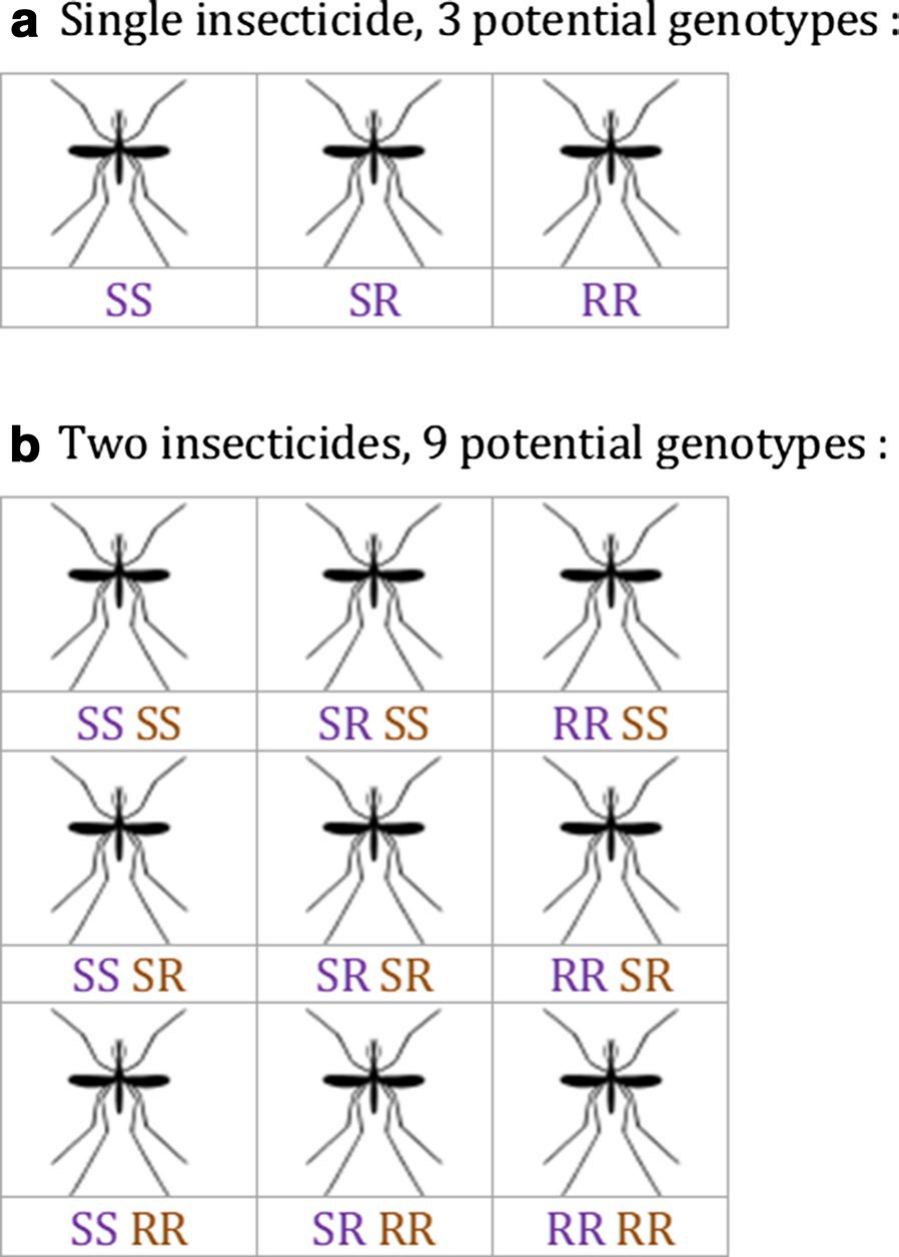
Potential resistance genotypes with **a** one and **b** two insecticides. An individual has two copies of the gene and each can be either resistant (R) or susceptible (S). Thus for one insecticide (**a**) individuals can either have both resistance genes (RR homozygous), both susceptible genes (SS) or one of each (SR, heterozygous). For two insecticides (**b**) there are 9 potential genotypes, here with the gene for one insecticide represented by the first two characters (purple) and the second insecticide by the final two characters (green). Thus RR SR represents an individual homozygous resistant to the first insecticide and heterozygous resistant to the second

The simulation represents a population of randomly mixing individuals using standard population genetic approaches to avoid the need to follow every individual. One gene or locus is represented for each insecticide. Each individual has two copies of the gene and there are two potential alleles per locus. Each allele confers either resistance or susceptibility to the insecticide. Thus individuals can either have both resistance genes (RR homozygous), both susceptible genes (SS) or one of each (SR, heterozygous). The combination of the two genes is termed the genotype. There are 3 genotypes when considering one insecticide and 9 genotypes (e.g. SSRR) when considering two insecticides (Fig. 1). Fitness is the main currency of the model, representing how much each genotype survives and reproduces. The fitness of the SS genotype in the absence of the insecticide is used as the reference genotype and assigned a value of 1. All other fitnesses are assumed to be less than 1, determined by alleles, exposure to the insecticide and other inputs that can be set in the model. These inputs include the effectiveness of the insecticide against SS genotypes, the dominance of the resistance allele and the ability of resistance to restore fitness of the RR genotype in the presence of the insecticide (see Table 1).

**Table 1:** Parameters influencing the development of insecticide resistance

Parameter	Description
1. Effectiveness	Proportion of susceptible (SS) insects killed by exposure to insecticide
2. Exposure	Proportion of insects exposed to insecticide
3. Resistance restoration	Ability of resistance (RR) to restore fitness of insects exposed to insecticide
4. Dominance of resistance	Sets fitness of heterozygous (SR) insects between that of SS and RR in presence of insecticide
5. Frequency	Frequency of resistance alleles within the population
6. Cost of resistance	Reduction in fitness of resistant (RR) insects in absence of insecticide
7. Dominance of cost	Sets fitness of heterozygous (SR) insects between that of SS and RR in absence of insecticide

**Fig. 2:**
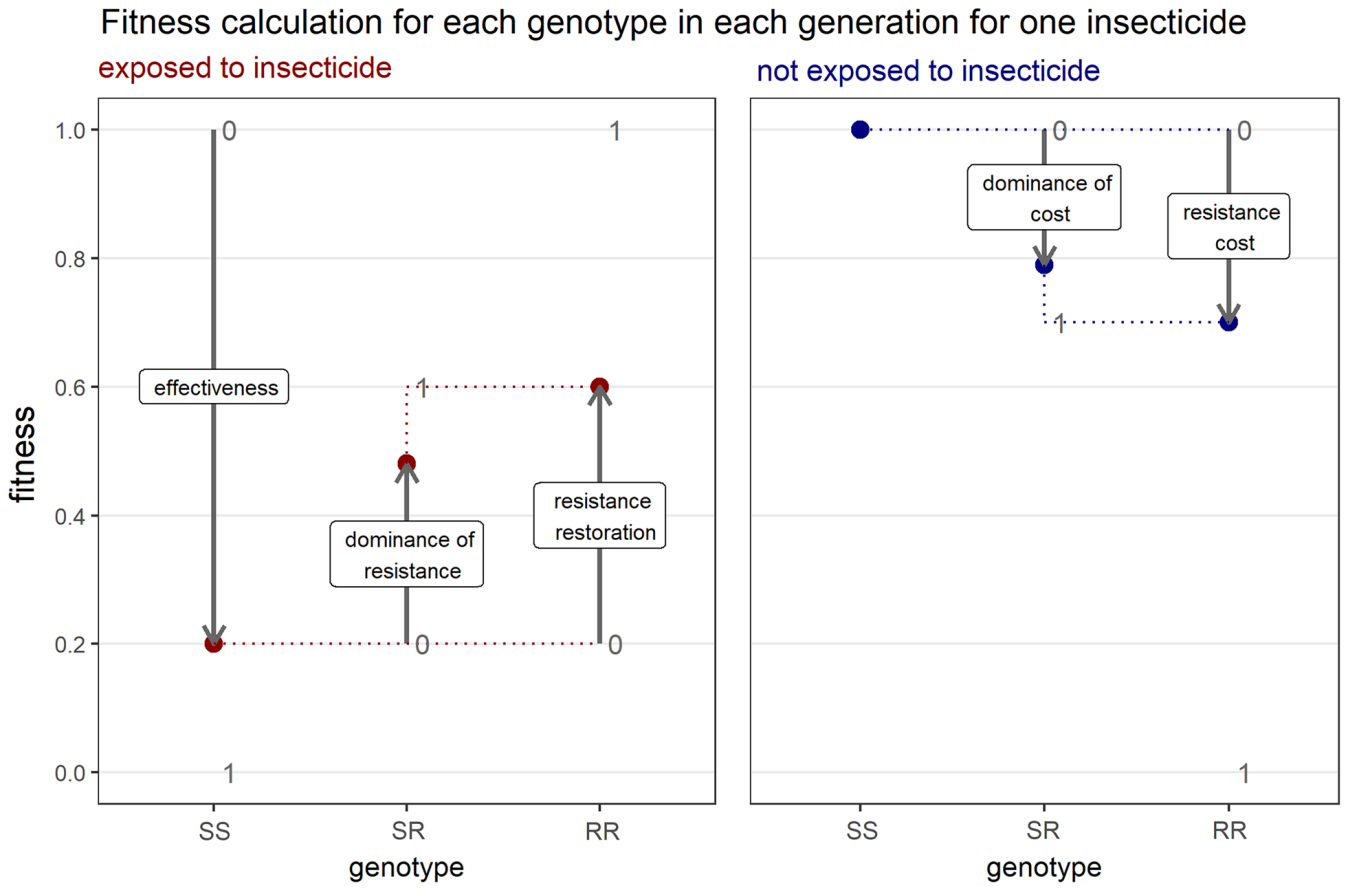
The effect of model inputs on the fitness of genotypes for a single insecticide. Fitness is shown on the y-axis and the different genotypes (SS, SR, RR) on the x axis. Firstly the exposure input determines the proportion of the population in the left and right panels (exposed and not exposed). For those that are exposed (left panel) insecticide effectiveness sets the fitness for SS, resistance restoration ‘restores’ a portion of the fitness for RR and dominance of resistance determines how the fitness for SR lies between that of SS and RR. For those that are not exposed, fitness of SS is set to 1 by definition, resistance cost determines the fitness of RR and again dominance of cost determines how the fitness for SR sits between that of SS and RR. In this example effectiveness = 0.8, resistance restoration = 0.5 which ‘restores’ half of the fitness lost due to the insecticide, dominance of resistance = 0.7 which sets the fitness of the SR closer to RR than SS. Resistance cost = 0.3 which reduces fitness in the absence of the insecticide from 1 to 0.7, and dominance of cost = 0.8 which sets fitness of SR close to RR

The calculation of fitness for each genotype when considering just a single insecticide is illustrated in Fig. 2. In the model itself, this is repeated for both insecticides and the fitnesses multiplied. Firstly, ‘exposure’ determines the proportion of the population in the left and right panels (exposed and not exposed). For those that are exposed (left panel) insecticide ‘effectiveness’ sets the fitness for SS and ‘resistance restoration’ restores a portion of the fitness for RR. A value of 1 for ‘resistance restoration’ would bring the fitness of RR back to 1, equivalent to the unexposed SS and meaning the RR genotype is completely unaffected by the insecticide. A value of 0 for ‘resistance restoration’ would lead to the RR having the same fitness as SS in the presence of the insecticide and effectively there would be no resistance. ‘Resistance restoration’ represents how much the RR genotype restores fitness in the presence of the insecticide.

‘Dominance of resistance’ determines how the fitness for SR sits between that of SS and RR for those that are exposed to the insecticide. With a value of 0 SR has the same fitness as SS (resistance is recessive) and a value of 1 SR has the same fitness as RR (resistance is dominant). For those that are not exposed, fitness of SS is set to 1 by definition and ‘Cost of resistance’ determines the fitness of RR. ‘Dominance of cost’ determines how the fitness for SR sits between that of SS and RR in the absence of the insecticide (Fig. 2).

The simulation proceeds through non-overlapping generations. In each generation selection is represented by multiplying genotype frequencies in the population by their relative fitness, the latter depending on their absolute fitness (Fig. 2) and level of exposure. This acts to make the fitter alleles more common over time. Separate sexes and standard sexual reproduction with recombination is included. There is also the potential to set exposure rates to be different for males and females to represent their different behaviours. Females are more likely to come into contact with insecticides on walls and nets when seeking to feed on humans compared to males that do not. Male exposure is only explored briefly here because a previous analysis indicated it was not a major factor determining differences between insecticide-use strategies [14].

So far this description just considers a single insecticide and associated resistance allele. In the model itself a second insecticide and resistance allele are included and overall fitness is calculated by multiplying the two results. This allows the model to represent populations exposed to two insecticides together in a mixture.

The model outputs the change in resistance allele frequencies over time measured in generations. The number of generations at which a resistance allele frequency of 50% is reached is recorded and termed ‘time-to-resistance’. Using a 50% threshold is consistent with previous modelling analyses (e.g. [11, 19]). Preliminary investigations showed that using other potential thresholds of 25 or 75% gave very similar results.

Three insecticide use strategies are investigated.

1. Single insecticide.
2. Two insecticides used in sequence, replacing the first with a second once the frequency of resistance alleles reaches a threshold of 50%.
3. Two insecticides used in a mixture (concurrently).

**Fig. 3:**
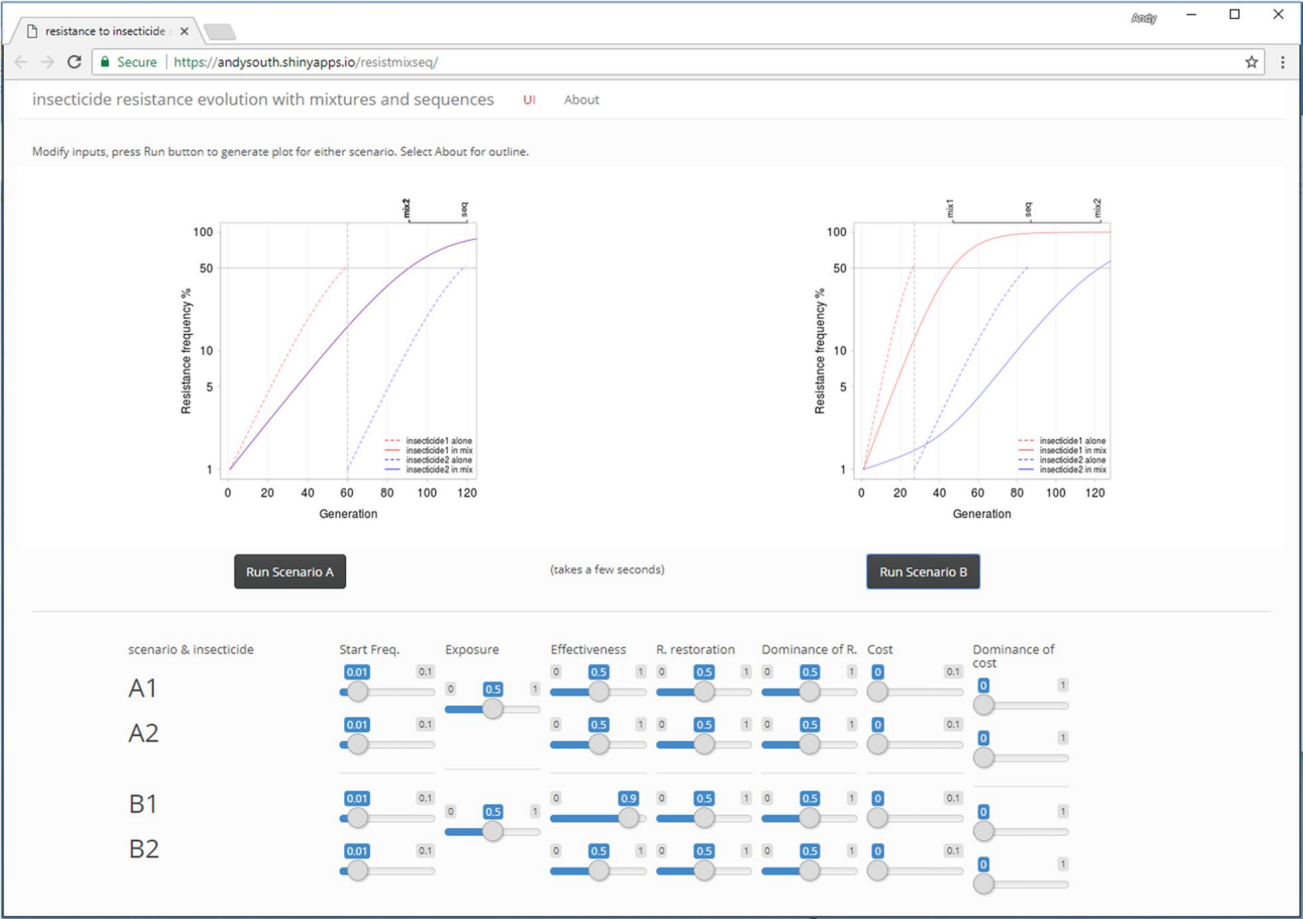
Screenshot of one online model user interface, accessible at: https://andysouth.shinyapps.io/resistmixseq. The user can modify values of the input parameters considered in this paper using simple sliders and run the model to get graphs of resulting resistance frequency over time. Two scenarios (A and B) can be run and the results viewed side by side. This makes it easy to explore the effect of changing individual inputs

A major aim of this paper is to use modern techniques to make the modelling accessible to a wider community. The model is implemented in R [20], the code is open-source and hosted on Github [21] including the code to generate both the figures and text of this paper relying on the packages knitr [22], shiny [23] and ggplot2 [24]. An on-line user interface is provided [25] (Fig. 3) accessing the same code and enabling readers with no coding experience to change inputs and run the model themselves.

## Results

The results start with a description of model runs with a single insecticide which are more straightforward and move on to two insecticides in sequences and mixtures.

### Single insecticide

**Fig. 4:**
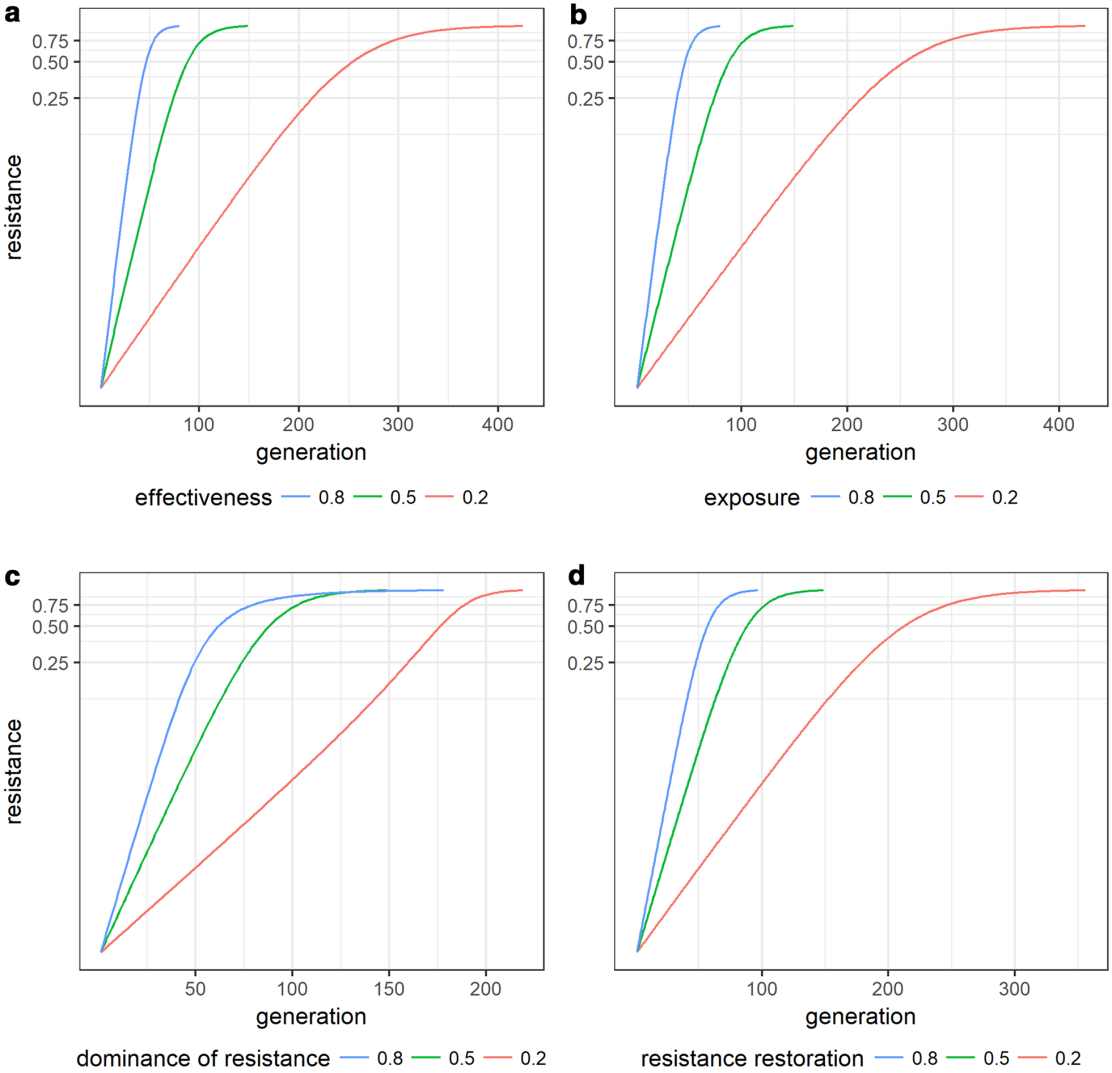
Single insecticide effects of effectiveness, exposure, dominance and resistance restoration on resistance frequency. **a** Effectiveness, **b** exposure, **c** dominance of resistance, **d** resistance restoration. Increasing any of the inputs (going from red to blue) results in shorter times-to-resistance. For each panel the chosen input was varied in isolation with the remaining inputs set to 0.5, except for starting frequency of resistance which was set to 0.01

**Fig. 5:**
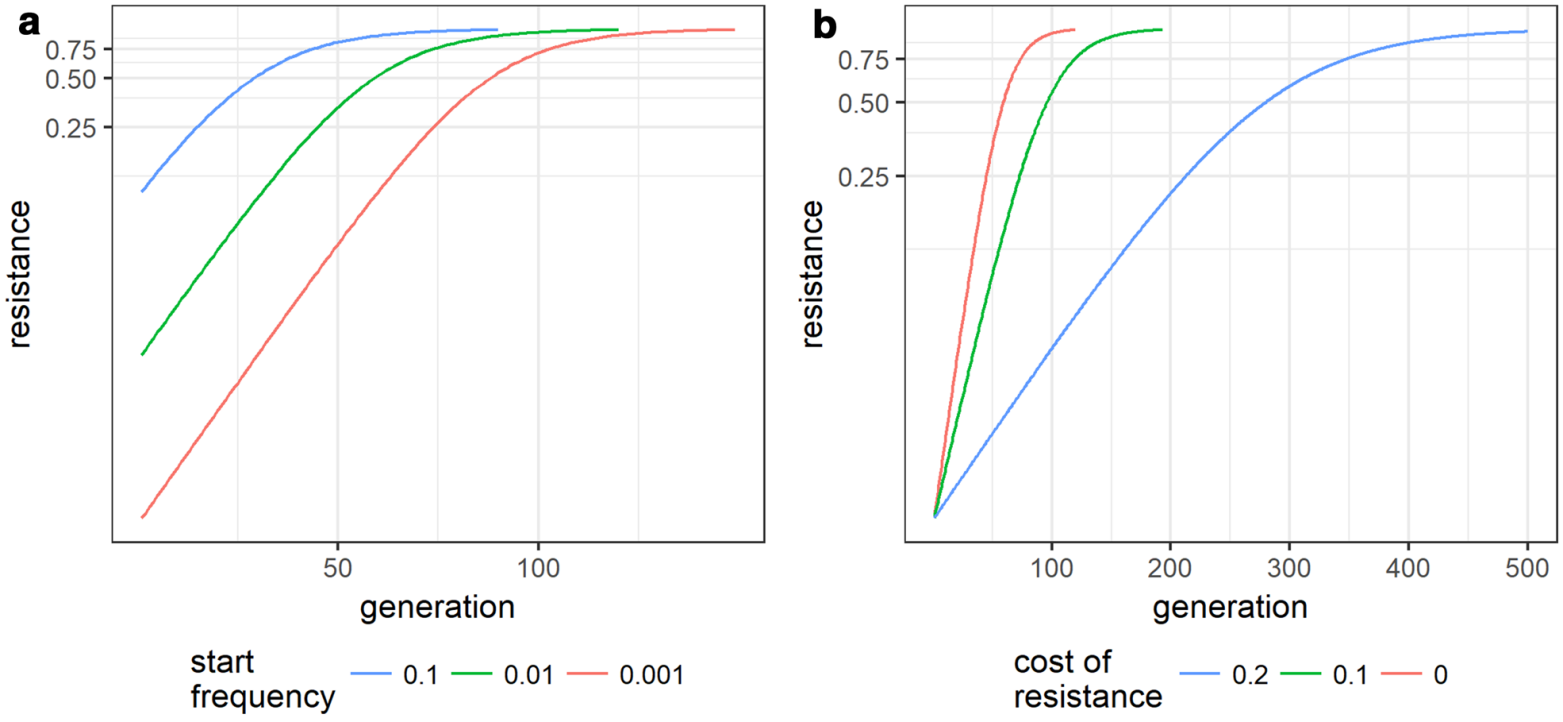
Single insecticide effects of starting frequency and cost of resistance on resistance frequency. **a** Starting frequency, **b** cost of resistance. Increasing the starting frequency decreases the number of generations taken to reach resistance thresholds. Increasing costs of resistance increases time-to-resistance

For single insecticide use, higher values of insecticide effectiveness, exposure, resistance restoration and dominance of resistance all resulted in faster resistance spread (Fig. 4a–d) and thus fewer generations to reach a resistance threshold of 0.5. This makes intuitive sense as all increase the impact of the insecticide and thus increase selection for resistance. Similarly, higher values of starting resistance frequency also resulted in shorter times to resistance thresholds (Fig. 5a) through the even simpler mechanism that a smaller change in frequency is required to reach the threshold.

Cost of resistance (Fig. 5b) had the opposite effect. Higher values led to a slower development of resistance. Again, this is intuitive because costs reduce the advantage of resistance and decrease selection. These effects of inputs on single insecticide use are summarized in Table 2.

**Table 2:** Effect of inputs on resistance when insecticides used singly or in sequence

Parameter to increase	Effect on resistance evolution	Mechanism
1. Effectiveness	Faster	Reduced fitness of SS and SR exposed to insecticide
2. Exposure	Faster	Reduced fitness of SS and SR overall
3. Dominance of resistance	Faster	Increased fitness of SR exposed to insecticide
4. Resistance restoration	Faster	Increased fitness of RR and SR exposed to insecticide
5. Frequency	Faster	Less change needed to reach resistance threshold
6. Cost of resistance	Slower	Reduced fitness of RR and SR not exposed to insecticide

### Two insecticides

**Fig. 6:**
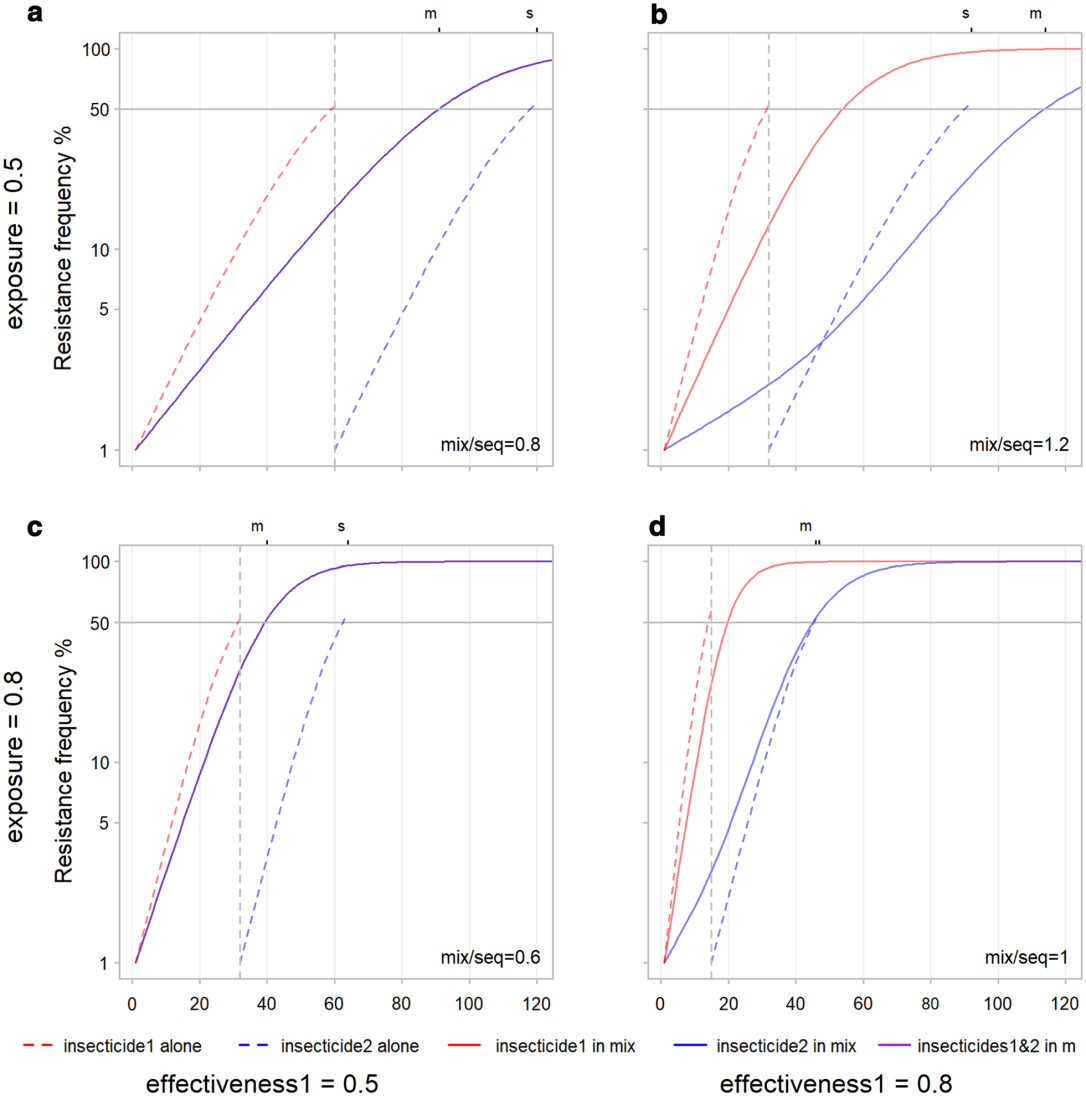
Influence of insecticide effectiveness and exposure on time-to-resistance for mixtures and sequences. Exposure to the insecticides is increased from row 1 (**a**, **b**) to row 2 (**c**, **d**). The effectiveness of one insecticide is increased from column 1 (**a**, **c**) to column 2 (**b**, **d**). On the upper X axis ‘s’ and ‘m’ indicate where the 50% resistance threshold is reached for the sequence and the mixture. In the lower right of each panel the ratio of time-to-resistance for mixture/sequence is shown rounded to 1 decimal place to give an indication of the relative performance of mixtures and sequences. **a** All control inputs equal at 0.5: time-to-resistance is longer for sequential use, **b** Effectiveness of insecticide 1 increased from 0.5 to 0.8: time-to-resistance is longer for the mixture, **c** exposure increased to 0.8: time-to-resistance is longer for sequential use, **d** effectiveness of insecticide 1 and exposure increased to 0.8: time-to-resistance equal for mixture and sequence. Increasing effectiveness increases times-to-resistance for mixtures and improves their performance relative to sequences. Increasing exposure decreases times-to-resistance for mixtures and reduces their performance relative to sequences

**Fig. 7:**
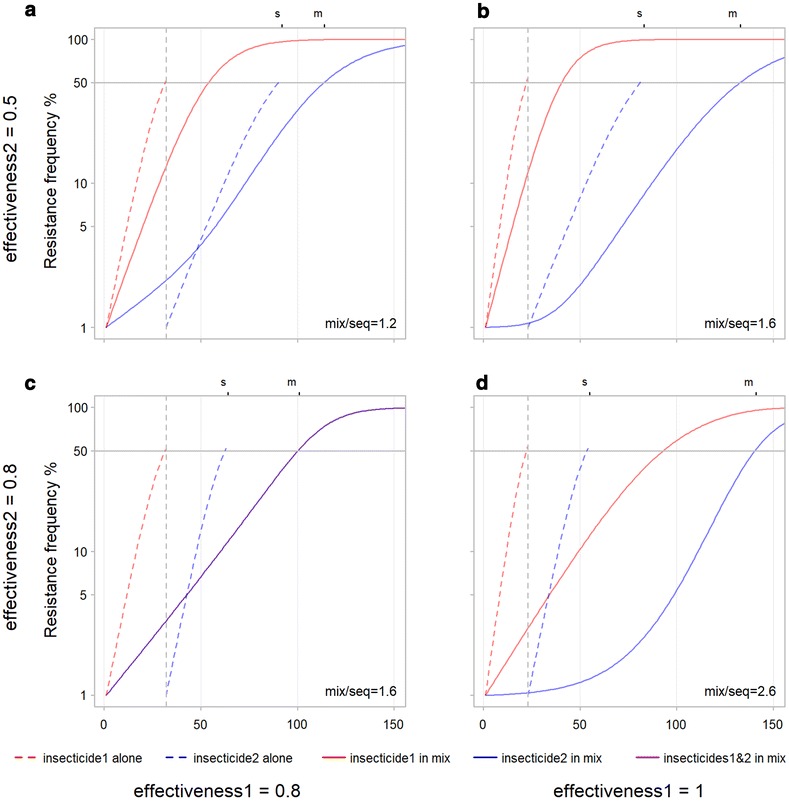
Influence of the effectiveness of both insecticides on time-to-resistance for mixtures and sequences. Effectiveness of insecticide 2 is increased from row 1 (**a**, **b**) to row 2 (**c**, **d**). The effectiveness of insecticide 1 is increased from column 1 (**a**, **c**) to column 2 (**b**, **d**). The ‘s’ and ‘m’ on the upper X axis and ‘mix/seq’ in the lower right of each panel stand for sequence and mixture and are explained in the legend to Fig. 6. **a** Effectiveness of insecticide 1 increased from 0.5 to 0.8, **b** effectiveness of insecticide 1 increased from 0.5 to 1, **c** effectiveness of insecticides 1 and 2 increased to 0.8, **d** effectiveness of insecticide 2 0.8 and of insecticide 1 1. With the effectiveness of at least one insecticide greater than or equal to 0.8, times-to-resistance are longer for the mixture in all scenarios

**Fig. 8:**
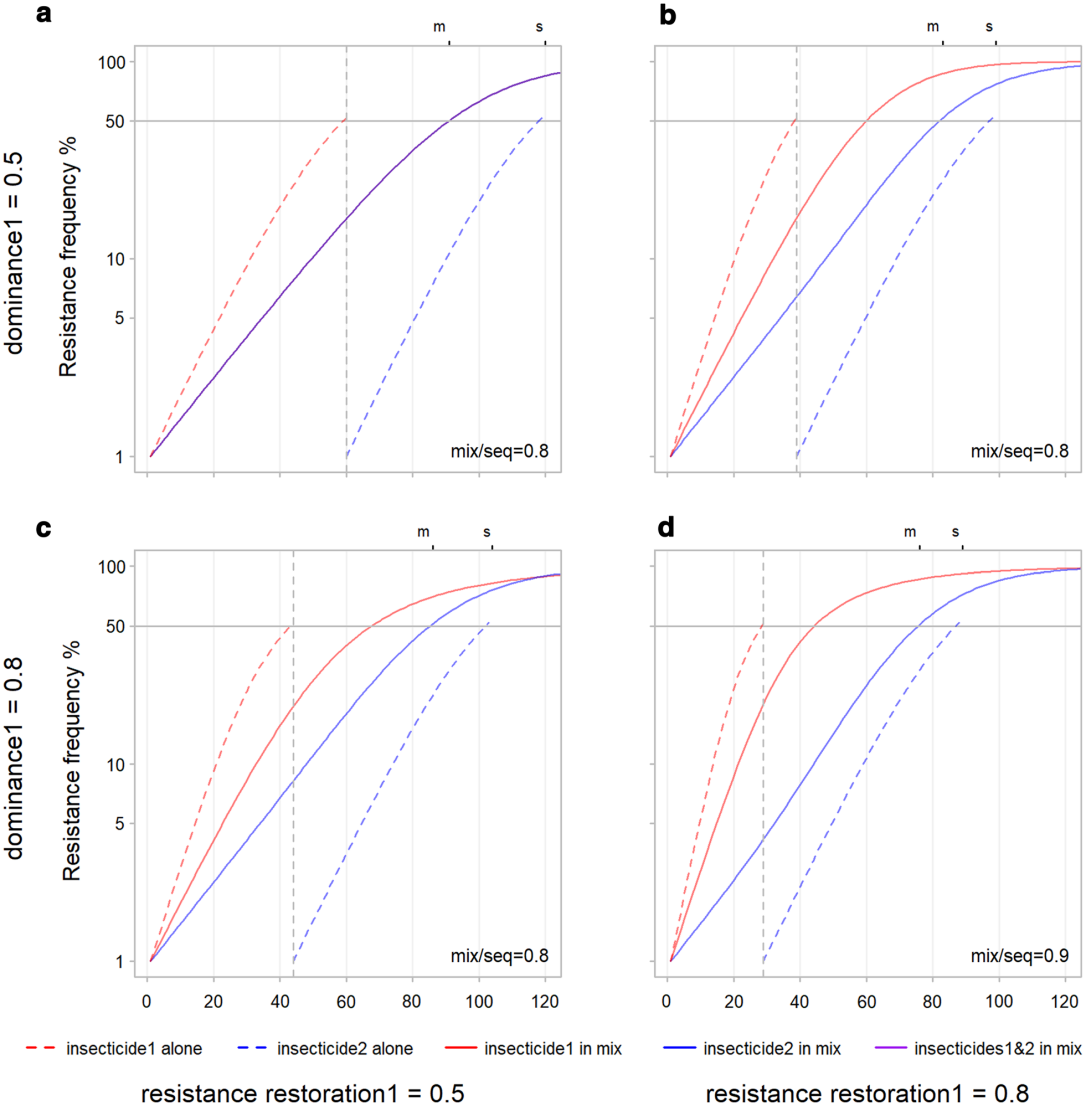
Influence of dominance and resistance restoration on time-to-resistance for mixtures and sequences. Dominance of the allele coding for resistance to insecticide 1 is increased from row 1 (**a**, **b**) to row 2 (**c**, **d**). Resistance restoration for the allele coding for resistance to insecticide 1 is increased from column 1 (**a**, **c**) to column 2 (**b**, **d**). The ‘s’ and ‘m’ on the upper X axis and mix/seq in the lower right of each panel stand for sequence and mixture and are explained in the legend to Fig. 6. **a** All control inputs equal at 0.5, **b** resistance restoration for insecticide 1 increased from 0.5 to 0.8, **c** dominance for insecticide 1 increased to 0.8, **d** resistance restoration and dominance for insecticide 1 increased to 0.8. Changing dominance and resistance restoration does not change the relative ordering of mixtures and sequences, time-to-resistance remains longest for sequences in all four scenarios

**Fig. 9:**
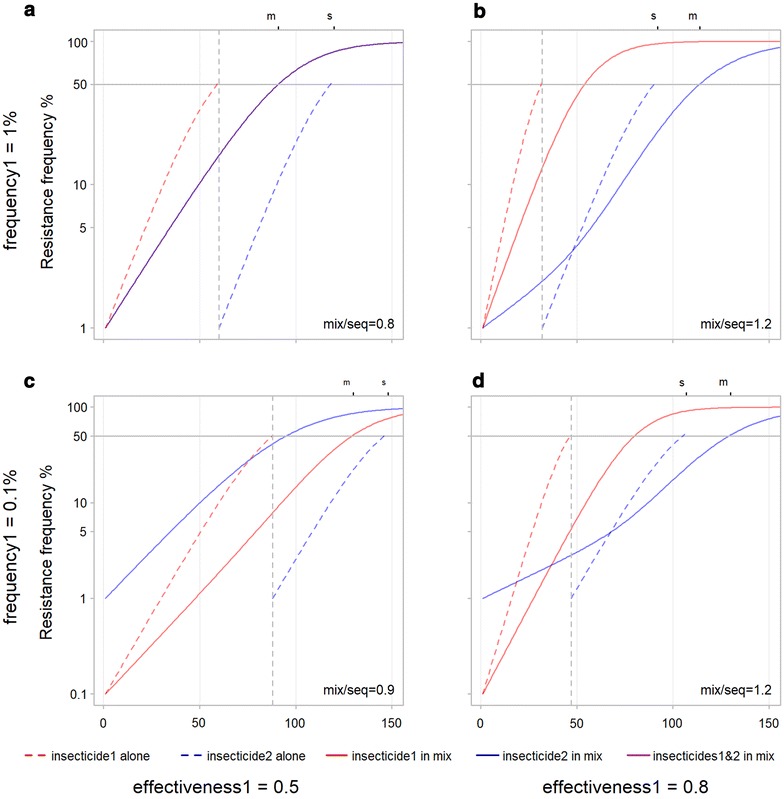
Influence of starting frequencies of resistance on time-to-resistance for mixtures and sequences. Starting frequency of the gene conferring resistance to insecticide 1 is decreased from row 1 (**a**, **b**) to row 2 (**c**, **d**). Effectiveness of insecticide 1 is increased from column 1 (**a**, **c**) to column 2 (**b**, **d**). The ‘s’ and ‘m’ on the upper X axis and mix/seq in the lower right of each panel stand for sequence and mixture and are explained in the legend to Fig. 6. **a** All control inputs equal at 0.5, starting frequencies of resistance at 0.01, **b** effectiveness for insecticide 1 increased from 0.5 to 0.8, **c** starting frequency of resistance for insecticide 1 decreased from 0.01 to 0.001, **d** effectiveness for insecticide 1 increased from 0.5 to 0.8 and starting frequency for insecticide 1 decreased from 0.01 to 0.001. In these scenarios the starting frequencies do not change better performance of sequences at low effectiveness and mixtures at high effectiveness

**Fig. 10:**
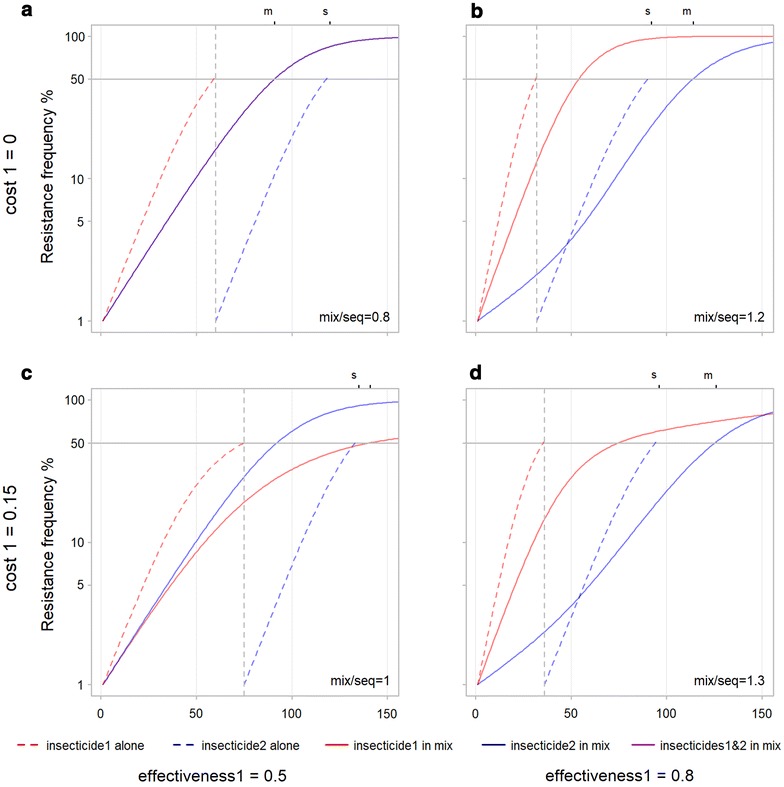
Influence of cost of resistance on time-to-resistance for mixtures and sequences. Row 1 (**a**, **b**) same as Fig. 6 with no cost of resistance, row 2 (**c**, **d**) cost of resistance set to 0.15 with dominance of cost set to 0.5. The ‘s’ and ‘m’ on the upper X axis and mix/seq in the lower right of each panel stand for sequence and mixture and are explained in the legend to Fig. 6. The increase in cost of resistance from **a** to **c** removes the advantage of sequence relative to mixture. The ratio of time-to-resistance for the mixture divided by that for the sequence goes from 0.8 to 1. Costs seem to favour mixtures in these plots. Also be aware that costs would lead to a decline in the frequency of resistance for the first insecticide in a sequence when it stops being used

When two insecticides are used, a comparison can be made between the relative performance of concurrent use in a mixture with sequential use. Each panel in Figs. 6, 7, 8, 9 and 10 compares mixture and sequence for a single combination of inputs. To help understand the mechanisms influencing this relative performance a single base scenario is used to demonstrate changing inputs individually. In this base scenario, effectiveness, exposure, resistance restoration, and dominance are set at 0.5, the starting frequencies of resistance to 0.01 and cost of resistance to 0. Resistance arises slower for sequential than mixture for this base scenario (Fig. 6a). Resistance to both insecticides in the mixture follow the same path and reach the threshold at the same time (as would be expected given that they have identical input parameters). When used in sequence the curve (dashed) for each insecticide individually is steeper than in the mixture but, because they happen one after the other, it takes longer for both to reach the resistance threshold (Fig. 6a). Thus, the time-to-resistance for the mixture divided by the sequence is less than 1 at 0.8.

### Insecticide exposure and effectiveness

Increasing exposure (the proportion of insects that come into contact with the insecticides) (Fig. 5c) decreases the time-to-resistance for both the sequential and mixture strategy. The effect on the mixture is greater so time-to-resistance remains longer for the sequence (the ratio decreasing from 0.8 to 0.6). In contrast keeping the exposure constant and increasing effectiveness (the proportion of SS insects that are killed by contact) of one of the insecticides (Fig. 6b) results in a longer time-to-resistance for the mixture relative to the sequence (and the ratio increasing from 0.8 to 1.2). The longer time-to-resistance for the mixture results from a slower increase in resistance for the less effective insecticide. Resistance to the less effective insecticide in the mixture increases slowly initially and then speeds up after the more effective insecticide has reached the resistance threshold. This points to a mechanism whereby the more effective insecticide initially slows the rate of evolution of resistance to the less effective one.

The result is that a more effective insecticide increases time-to-resistance when used in a mixture (compare the solid lines in Fig. 6a, b). This is opposite to what happens when used in sequence (compare the red dashed line in Fig. 6a, b) and in isolation (Fig. 4a), when more effective insecticides shortened times-to-resistance.

In summary increasing effectiveness can favour mixtures moving from column 1 to 2 in Fig. 6, and increasing exposure can favour sequences moving from row 1 to 2 in Fig. 6. Reduced male exposure with respect to female, as would be expected for male mosquitoes not seeking blood meals, had a similar effect to reducing overall exposure.

Increasing the effectiveness further of either or both insecticides increases the performance of mixtures over sequences in terms of time-to-resistance. Figure 7a uses the same inputs as Fig. 6b. From this scenario a higher effectiveness for insecticide 2 (Fig. 7c) or insecticide 1 (Fig. 7b) or both (Fig. 7d) all result in a greater positive difference in time-to-resistance for mixture over sequence. This again points to how more effective insecticides can slow resistance to another when used in a mixture.

### Resistance restoration and dominance

Increasing resistance restoration or its dominance decreases time-to-resistance for both sequences and mixtures (Fig. 8) in the same way that it did for sole use (Fig. 4). The result is that increasing either or both of dominance and resistance restoration (Fig. 8b–d) does not change the relative time-to-resistance for mixtures and sequences from that in the base scenario (Fig. 8a) where it takes resistance longer to develop for the sequence. So, unlike effectiveness and exposure, the levels of resistance restoration and dominance do not effect whether a mixture or a sequence are likely to be favoured.

### Starting frequencies of resistance

Changing the starting frequency of resistance had a similar effect on time to resistance for both sequences and mixtures and thus had little effect on their relative performance. For example, taking the base scenario and reducing the starting frequency of one resistance allele did not change from sequence being favoured (compare Fig. 9c to a). Similarly taking a scenario in which time-to-resistance is longer for a mixture (Fig. 9b) and decreasing the starting frequency of that resistance allele did not change the fact that the mixture was favoured (Fig. 9d). In these analyses, starting frequencies of resistance were set relatively high at 0.01 or 0.001. In a more detailed sensitivity analysis [14] the lower limit for starting frequencies was set to 0.0001 and still it was not a key parameter in determining the relative performance of mixtures and sequences.

### Costs of resistance

Increasing the cost of resistance to one insecticide in a mixture leads resistance to that insecticide to evolve more slowly (compare the solid red line to the solid blue line in Fig. 10c, the red line is for the resistance with the higher cost). The resistance with the higher cost also increases more slowly in the mixture relative to when it is used in sequence (compare the solid red line to the dotted red line in Fig. 10c). The greater effect of cost on mixtures means the advantage of sequences over mixtures in Fig. 10a (and Fig. 6a) is removed in Fig. 10c. With costs set to 0 time-to-resistance for mixture divided by sequence is 0.8 in Fig. 10a and 1 in Fig. 10c. At higher insecticide effectiveness the benefit of mixture over sequence is improved as seen by comparing Fig. 10d to b.

A summary of the effects of inputs on the evolution of resistance for mixtures is given in Table 3 and for the difference between mixtures and sequences in Table 4. The mechanisms identified in the tables are covered further in the discussion.

**Table 3:** Effect of inputs on resistance when insecticides used in a mixture

Parameter to increase	Effect on resistance evolution	Mechanism
1. Effectiveness	Slower	One insecticide reduces the fitness of individuals resistant to the other thereby reducing selection pressure for the other
2. Exposure	Faster (but less than for insecticide used alone)	Reduced fitness of individuals susceptible to one insecticide increases selection pressure for that resistance. However at the same time selection pressure is reduced by reduced fitness of resistant individuals caused by the other insecticide
3. Dominance of resistance	Faster	Increased fitness of heterozygotes
4. Resistance restoration	Faster	Increased fitness of resistants
5. Frequency	Faster	Less change needed to reach resistance threshold
6. Cost of resistance	Slower	Reduced fitness of resistants in absence of insecticide

**Table 4:** Effect of inputs on the difference between mixture and sequential use

Parameter to increase	Increase favours mix or sequence	Mechanism
1. Effectiveness	Mixture	Higher effectiveness gives faster resistance for sequence and slower resistance in mixture
2. Exposure	Sequence	Higher exposure gives faster resistance for sequence and mixture but the greater effect on mixture favours sequence
3. Dominance of resistance	Neither	Higher dominance gives faster resistance in both sequences and mixtures such that the difference between them is not changed
4. Resistance restoration	Neither	As for dominance of resistance
5. Frequency	Neither	As for dominance and resistance restoration
6. Cost of resistance	Mixture	Higher costs slow the evolution of resistance more in a mixture than when used in sequence (but with higher costs in a sequence there is a greater chance for resistance levels to decline for the insecticide not being used)

### Linkage disequilibrium

Positive linkage disequilibrium is said to exist when the frequency of two alleles at different loci is higher than expected if they were just selected individually. It has previously been suggested [1, 11] that using an insecticide to which there was existing resistance in a mixture with a new insecticide with little resistance could lead to more rapid evolution of resistance to the latter through linkage disequilibrium. This paper includes two scenarios with a mixture of a new insecticide and one with pre-existing (higher) resistance (Fig. 9c, d). In both cases resistance to the newer insecticide with lower starting resistance (in red) increases faster when it is used alone (red dotted) than when it is used in a mixture (red solid). This suggests that linkage disequilibrium does not restrict the potential benefits of mixtures in all situations. These results support the statement in GPIRM [1] that: “Even though the risk for linkage disequilibrium exists, mixtures may still be the most attractive IRM tool …”. The dynamics of LD is too complex to discuss in detail here but interested readers can find more discussion in [1, 11, 14].

### Setting model inputs from field data

The main model inputs quantifying properties of the mosquitoes and insecticides can be derived from data on the relative survival of the different genotypes (RR, SR, SS) as described in Table 5.

**Table 5:** Equations to calculate model input parameters from field data (derived from [14] see Fig. 2)

Parameter	Calculation
1. Effectiveness	1—SSfit in presence of insecticide
2. Exposure	estimated
3. Resistance restoration	(RRfit-SSfit)/effectiveness in presence of insecticide
4. Dominance of resistance	(SRfit-SSfit)/(RRfit-SSfit) in presence of insecticide
5. Frequency	estimated
6. Cost of resistance	1—RRfit in absence of insecticide
7. Dominance of cost	(SRfit-RRfit)/(RRfit-SSfit) in absence of insecticide

Where RRfit, SRfit, SSfit are fitnesses of genotypes

Exposure, the proportion of mosquitoes that are exposed to the insecticide, is a property of the location and will depend on the use of nets or IRS and mosquito behaviour. The frequency of resistance alleles can be measured, although when at low levels a large number of mosquitoes would need to be sampled to detect low frequencies.

As an example of making these calculations, here *Anopheles gambiae* data are extracted for pyrethroid (Alpha-cypermethrin) resistance associated with the *kdr* mutation [26] and carbamate (Bendiocarb) resistance associated with the *ace1* mutation [27]. This is not intended to be a definitive prediction of resistance evolution, sample sizes for some genotypes are low so fitnesses are approximate. It is intended to demonstrate how models of resistance evolution can be set to field data and hence bring the two approaches closer together.

Data were provided as numbers alive and dead by genotype in both publications. These data are used to calculate genotype-specific survival values as an indication of fitness. The non-exposed control data for the pyrethroid [26] are used to estimate the cost of resistance and to rescale the fitness estimates on a scale of 0–1 where 1 is the fitness of the unexposed SS (i.e. all pyrethroid survivals are divided by that of the unexposed SS genotype, 0.83). In the absence of unexposed data for the carbamate [27], it was assumed there were no costs of resistance and that survival of the non-exposed SS would be 1. These estimates were used to calculate the values of model inputs as shown in Table 6.

**Table 6:** Model input parameters calculated from field data

Field data	Pyrethroid, Kdr [26]	rescaled pyrethroid, Kdr [26]	Carbamate, Ace1 [27]
Exposed survival RR	0.83	1	0.84
Exposed survival SR	0.72	0.87	0.56
Exposed survival SS	0.59	0.71	0.02
Unexposed survival RR	0.67	0.81	–
Unexposed survival SR	0.50	0.60	–
Unexposed survival SS	0.83	1	–
Calculated model inputs			
Effectiveness	0.41	0.29	0.98
Resistance restoration	0.60	1	0.84
Dominance of resistance	0.52	0.54	0.66
Cost of resistance	–	0.19	–
Dominance of cost	–	1^a^	–

^a^For the pyrethroid the unexposed survival of the SR (0.5) was less than that for the RR (0.67) This indicates underdominance which is biologically unlikely for unexposed genotypes so dominance was set to 1 so that SR and RR genotypes have the same fitness costs. The authors indicated that surprisingly high mortality in the control huts may have been due to rough handling [26] and this could contribute to the unexpected values here

**Fig. 11:**
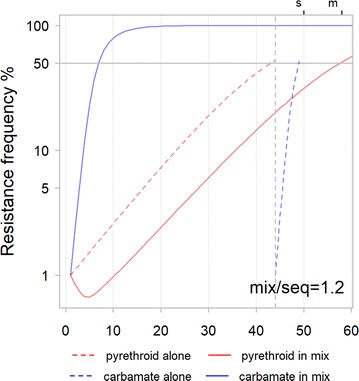
Time-to-resistance for a mixture and sequence using inputs derived from field studies (as outlined in Table 6). Alpha-cypermethrin (pyrethroid) is shown in red and Bendiocarb (carbamate) is shown in blue. Exposure is set to 0.8 and starting frequencies to 0.01. Resistance to the carbamate (blue) increases very quickly in both the mixture and sequence due to its high effectiveness. Resistance to the pyrethroid (red) increases more slowly due to low effectiveness and high cost of resistance. In a mixture the resistance frequency of the pyrethroid even declines when resistance to the carbamate is low. In this illustrative example resistance to both insecticides takes longer to evolve for a mixture than a sequence

The results of using these calculated inputs in one model example are shown in Fig. 11 (bearing in mind that this is a demonstration exercise for setting model parameters from field data and not a definitive prediction for whether using a pyrethroid and a carbamate in a mixture or sequence is likely to be preferable).

## Discussion

Resistance responds to insecticide use in the model in ways that can be explained mechanistically through the process of selection. This mechanistic explanation can help develop a more robust understanding of how resistance is expected to evolve in the field. It is helpful to start by explaining the evolution of resistance to a single insecticide, which is relatively straightforward, and move on to explaining the effect of two insecticides in sequences and mixtures.

When single insecticide use was investigated (Figs. 4, 5) higher values of parameters that increased the selective advantage of resistance all led to more rapid evolution of resistance, whereas higher values for cost led to slower evolution of resistance (Fig. 5b). Mechanisms explaining the evolution of resistance to single insecticide use, are summarised in Table 2. Referring to Fig. 2 can help explain these responses. Exposure and effectiveness act by decreasing the fitness of SS and SR more than RR, dominance of resistance acts by increasing fitness of SR and resistance-restoration acts by increasing fitness of RR and SR. Cost acts by decreasing the fitness of RR and SR among those mosquitoes not exposed.

### Two insecticides

Responses to input parameters are different when two insecticides are used either in sequence or in mixtures as compared to when used alone. These differences can help us to understand the dynamics of resistance evolution. In each panel of Figs. 6, 7, 8, 9 and 10 the solid lines, indicating that an insecticide is being used at the same time as another in a mixture, are always shallower than the same colour dashed line indicating the insecticide is being used in isolation as part of a sequence. The shallower curves for the mixture can be explained by each insecticide killing individuals that are resistant to the other insecticide. Because resistant individuals are being killed, the selection pressure for that resistance is lower. Thus, each insecticide reduces the selection pressure for resistance to the other and could be said to ‘protect’ the other. This protection means that evolution of resistance to one insecticide is always slower when it is used in a mixture than when used alone. This protection has been termed multiple intra-generational killing [10] because individuals have the potential to be killed by more than one insecticide.

Whilst this protection ensures that resistance to each insecticide evolves more slowly in a mixture, it does not guarantee that a mixture strategy is preferable to a sequence once the resistance to both insecticides is taken into account. In a mixture, selection pressure is applied by both insecticides at the same time, whereas in a sequence there is no selection for the insecticide not being used. Resistance can evolve more slowly for both insecticides in sequential use, despite being faster for each, because they occur one after the other. In Fig. 6a, it takes 60 generations for resistance to reach the threshold for each insecticide in the sequence, and 90 when they are applied together in the mixture, so the sequence is still favoured because 2 × 60 is greater than 90.

This advantage of sequence over mixtures can be removed by increasing the effectiveness of one of the insecticides. Increasing the effectiveness of one insecticide (Fig. 6b) decreases the total time for the sequence from 120 to 90 and increases that for the mixture from 90 to 115 thus changing the order and favouring the mixture. The mechanism for this change can be seen by comparing the shape of the curves in Fig. 6a, b. In the mixture, resistance to insecticide 1 (with the increased effectiveness) rises at a similar speed in both figures. However, the increased effectiveness of insecticide 1 causes resistance to insecticide 2 to increase more slowly initially in the mixture. Once insecticide 1 reaches resistance of around 50% at around 50 generations, the curve for insecticide 2 becomes steeper. Once the first insecticide reaches the resistance threshold of 50% it kills fewer individuals that are resistant to the second insecticide, it loses its protective effect and thus stops slowing the rise in resistance to the second insecticide. A similar effect is visible when the effectiveness of both insecticides are increased (Fig. 7c). The identical resistance curves for each insecticide in the mixture are shallower than at the lower effectiveness (Fig. 6a) because more individuals resistant to each insecticide are being killed by the other insecticide. In this case, the ‘protection’ given to both insecticides by the other declines at the same rate so there is no change in slope as there is in Fig. 6b. In both cases increasing the effectiveness of insecticides increases their protective effect when used in mixtures and favours mixtures over sequences.

Figure 6b also demonstrates that when two insecticides have a different effectiveness in a mixture, other parameters being equal, resistance will be expected to increase faster to the more effective insecticide (the red curve in this case). The more effective insecticide prompts both (a) higher selection pressure for its own resistance and (b) greater ‘protection’ reducing the rise in resistance to the other insecticide. The more effective insecticide has a faster evolution of resistance than the less effective one but this is not caused by the presence of the less effective one. Indeed, the presence of the less effective one is still slowing the evolution of resistance to the more effective one, as indicated by the red solid curve for use in the mixture being shallower than the red dashed curve for use alone as part of the sequence.

### Difference between exposure and effectiveness

It seems initially counter-intuitive that whereas increasing effectiveness slows the development of resistance for mixtures, increasing exposure speeds it up. Increasing the exposure to both insecticides results in a decrease in time-to-resistance (from 90 generations in Fig. 6a to 40 generations in Fig. 6c) where increasing the effectiveness of both insecticides by the same amount results in a slight increase (from 90 in Fig. 6a to 115 in Fig. 6b). This contrasts with the identical effects that exposure and effectiveness have on a single insecticide (Fig. 4a, b) and on insecticides in sequence (the red dashed lines are the same in Fig. 6b, c). This difference, that exposure reduces times-to-resistance for a mixture and effectiveness increases it, was unexpected. The most likely explanation is that increasing exposure increases overall selection pressure and lowers time to resistance, whereas increasing effectiveness increases mutual protection of the insecticides in a mixture and lowers the speed at which resistance evolves.

### Costs of resistance

Fitness costs of resistance have been widely reported (review in [28]), but it has been suggested that few of these are directly relevant to control failure in the field [29]. Because of this likely rarity of costs, they are not included in most of the analyses shown here (with the exception of Fig. 10). Costs of resistance favour mixtures in these analyses because the evolution of resistance is slowed more by cost in a mixture than in a sequence (compare the red solid lines for a mixture to the red dotted line for a sequence in Fig. 10c, d). It makes sense that the protective effect of the other insecticide in a mixture combines with the cost of resistance to reduce selection pressure more than in a sequence.

The resistance curves for two insecticides in a mixture that differ only in their cost of resistance, diverge more as the frequency of resistance increases (Fig. 10c). This makes intuitive sense, as the frequency of resistance genes increases the impact of fitness costs associated with those genes also increases. Thus, differences due to cost are less noticeable at low resistance frequencies and become more noticeable at higher resistance frequencies. Thus, if a lower resistance threshold was used cost would have less of an effect and if a higher resistance threshold was used cost would have more of an effect. If costs are set even higher than set here (e.g. above 0.2) they can maintain resistance frequencies below 1 or lead to their decline. However, such high costs are not expected to occur in operational settings [29].

Declines in resistance frequencies are likely to occur when there are costs of resistance and the insecticide is not being used [29]. Thus, the frequency of resistance for the first insecticide in a sequence, after it has been replaced, may decline to a level where the first insecticide becomes operationally useful again. Consequently, a repeated sequential strategy could offer benefits over mixtures not considered here. To quantify these benefits it would be necessary to use a more detailed metric than the time-to-resistance (the number of generations until resistance thresholds are first reached). Other measures for quantifying resistance over time can be imagined, for example the mean resistance or proportion of generations below a resistance threshold.

### Using field derived inputs

Model behaviour when using the field derived inputs (Fig. 11) can be explained using the understanding developed from the preceeding runs. Resistance to the carbamate (blue) rises quickly partly because it has high effectiveness (0.98). Resistance to the pyrethroid (red) increases more slowly due to its much lower effectiveness (0.29) and high cost of resistance. In the mixture, the resistance to the pyrethroid declines at the start when resistance to the carbamate is low. This illustrates the protective effect of the insecticide with the higher effectiveness. That protective effect causes the mixture to out-perform the sequence in this example. Indeed, if exposure is set lower to 0.5 then resistance to the pyrethroid declines further and the resistance threshold is not reached. This is a combination of the low effectiveness creating a low selection pressure for the resistance and the high cost creating a selection pressure against it. The low effectiveness and high cost are partly due to the surprisingly high mortality in unexposed mosquitoes in the field data [26].

### Consistency with previous work

The predicted responses of resistance presented here can be put into the context of previous work and recommendations. A recent review [10] found that 8 out of 10 empirical studies and 11 out of 14 modelling studies favoured mixtures over sequences. In the 3 other modelling studies the relative performance of mixtures and sequences were dependent on the values of other inputs (as also shown here).

The review concluded that the advantage of mixtures is greatest when seven other conditions are satisfied [10]: (i) rare starting resistance, (ii) independent loci (i.e. no cross resistance), (iii) high recombination, (iv) high susceptible mortality, (v) resistance is recessive, (vi) similar persistence of insecticides, and vii) some of population remains untreated. Their points (iv) and (vii) agree with our results that mixtures are favoured by high effectiveness and low exposure. Some of the other conditions, notably (i) the starting frequency of resistance and (v) the dominance of resistance did not alter whether mixtures or sequences were favoured in our model [14].

Earlier modelling work has made sometimes contrasting predictions and these can be explained by the details of their structure and input choices. One model predicted that “the use of mixtures is always more effective in delaying the onset of resistance often by many orders of magnitude” [12]. That model assumed that SS genotypes were always killed, RR always survived, and that a constant 10% of mosquitoes escaped the insecticide. This would be represented in the model presented here by a very high ‘insecticide effectiveness’ of 1, a ‘resistance restoration’ of 1 and an ‘exposure’ of 0.9. This result is consistent with our prediction that the very high effectiveness is likely to favour mixtures over sequences (e.g. see Fig. 7d and Table 4).

A subsequent model lead to the following more pessimistic conclusion about the potential of mixtures: “As a result of incomplete coverage and residue decay, the mortality of susceptible homozygotes is rarely consistently high enough for pesticide mixtures to be effective” [30]. This “mortality of susceptible homozygotes” is equivalent to our insecticide effectiveness. Again this points to the importance of effectiveness and the question of whether the effectiveness levels needed to make mixtures better are attainable operationally. This earlier model [30] (Fig. 3) is also consistent with that presented here in showing that decreasing exposure favours mixtures over sequences.

### Caveats

There is evidence of the involvement of multiple genes in resistance mechanisms [16, 31, 32]. The model presented here, in common with almost all previous ones [33], assumes a single ‘resistance’ gene per insecticide. Target-site resistance has been shown to be mostly determined by single genes, and control failures in general have mostly been attributed to the effect of single major genes [13, 16, 31]. One view is that control doses of insecticides in the field are likely to lead to the selection of single major genes whereas the lower doses used to select laboratory strains are more likely to lead to polygenic resistance [16]. There is also evidence that low insecticide doses in agriculture have led to polygenic resistance [32]. To represent polygenic resistance a different ‘quantitative genetic’ modelling approach would be required [34, 35]. A recent review of 187 models of the evolution of resistance [33] did not include any that represented quantitative multiple gene resistance. However there are a few published studies applying a quantitative genetic approach to assessing insecticide use strategies in the context of polygenic resistance [34, 36, 37]. If it becomes clearer that polygenic resistance is an issue for vector control then there will be a need for appropriate quantitative genetic models and a comparison of those to single gene approaches like this one.

### Poor implementation

One aspect of the results presented here can seem to contradict published advice and has been questioned when the results of this model have been presented. This model predicts that lower insecticide effectiveness or exposure will lead to slower spread of resistance in sole use and sequences (Figs. 4, 6, Table 2). This is consistent with expectations that lower effectiveness and exposure would lead to lower mortality of susceptibles and thus lower selection pressure for resistance [30]. This can seem to contradict recommendations that poor implementation of insecticide interventions can promote the development of resistance. e.g. from [5] “Certain pest control practices have consistently been shown to exacerbate the loss of susceptible pest populations and the development of resistance. These include: … - the use of application rates that are below or above those recommended on the label, - poor coverage of the area being treated…”.

Poor implementation would likely cause lower effectiveness of, and exposure to, insecticides, so how can this increase the potential for resistance when our model suggests it would be decreased? There are two main potential explanations for this apparent difference, (a) dominance and (b) polygenic resistance. The effect of dominance is that poor application can reduce the mortality of heterozygotes (SR) thus effectively increasing the dominance of the resistant allele and increasing its rate of evolution [5, 38, 39] see Fig. 1 of [14]. The results presented here are consistent with this, showing that increasing dominance of resistance leads to faster spread of resistance (Figs. 4c, 8). So, the effect of poor implementation of an insecticide intervention on resistance could be either positive or negative depending on the relative effects on dominance of the resistance gene, insecticide effectiveness and exposure. Similarly, the effects of poor application on the relative benefits of mixtures and sequences will depend on these trade-offs following the results presented here that higher effectiveness favours mixtures, higher exposure favours sequences and higher dominance favours neither (Table 4). More information on how the mortality of different genotypes (susceptible, resistant and heterozygous) is affected by poor application rates or insecticide degradation over time would help make this clearer. Secondly, if resistance is predominantly polygenic rather than monogenic poor implementation is likely to promote resistance by allowing the build up of many genes of small effect [32]. Indeed modelling work from 15 years ago [36, 37] proposes an alternation of low and high doses with the low doses limiting the evolution of monogenic resistance and the high doses limiting that of the polygenic resistance. More information on the relative importance of monogenic and polygenic resistance for vector control would help make this clearer.

Experience from agriculture where single interventions have been associated with a rapid development of resistance have led for calls for a more Integrated Vector Management composed of a series of partially effective tools [40]. It is suggested that such an integrated approach is more sustainable and ‘evolution-proof’ [40]. A similar combining of interventions has been advocated to cover mosquitoes exhibiting different behaviours [41]. Mosquitoes with different behaviours e.g. feeding outdoors and/or on animals are likely to result in lower exposures to insecticides in nets or sprayed on walls. This lower exposure could favour mixtures over sequences as indicated here. In both cases this modelling approach could be used to investigate implications for the development of resistance.

Even if the assumption that single genes are responsible for control failures is correct there is still considerable other uncertainty around the development of insecticide resistance in operational field settings [2]. This model is able to represent much of that uncertainty, yet in this paper the exploration has been restricted to a limited region of parameter space. Most of the inputs have been held constant at intermediate values while modifying single inputs in isolation. This is a deliberate tactic to develop understanding and communicate the key mechanisms. There is the potential that the system may behave differently in the field. However, the results presented here are supported by an earlier analysis where 10,000 unique scenarios were run, varying more inputs [14]. This previous analysis also highlighted the relative importance of insecticide effectiveness and exposure in determining the relative performance of mixtures and sequences.

In addition, an on-line user interface to the model (Fig. 3) [25] is provided, enabling readers with no coding experience to investigate model behaviour beyond that shown in this paper. The user-interface allows inputs to be set for two scenarios and results to be viewed side by side. For example, a higher insecticide effectivenesses could be set to see how this favours mixtures over sequences (Fig. 3) or exposure levels could be increased to see how this favours sequences.

There are other model behaviours that can be changed by modifying model inputs within the computer code. For example, here it is assumed that insecticide interaction in a mixture is multiplicative, e.g. if the probability of surviving exposure to insecticide A alone is 0.3 and of surviving insecticide B alone is 0.2, then the probability of surviving exposure to a mixture of A and B is 0.3 * 0.2 = 0.06. This is consistent with recent work on aphids [42], although that did also indicate some synergistic effects. Modifying model inputs would allow such synergistic or antagonistic effects of mixtures to be incorporated, i.e. that insecticides used together perform better or worse than when used separately. This would allow the model to represent cross-resistance where a resistance gene provides resistance to more than one insecticide. Similarly, behavioural resistance is likely to offer resistance against more than one insecticide [1], but until there are more data on whether behavioural resistance is genetic and heritable, representing it in this way is not advisable.

In addition to these uncertainties regarding resistance it is important to remember that this model examines solely the evolution of resistance and not the control of mosquitoes or malaria. Different insecticide use strategies, as well as affecting the evolution of resistance, will also influence both the numbers of vectors and their propensity to transmit the disease [43]. There are also potential knock-on effects of resistance itself; reduced vector transmission of disease (e.g. by reduced lifespan of the vector) or increased transmission (e.g. by increasing the susceptibility of the vector to the disease) [43]. This model can explore when a mixture strategy may be favoured over a sequence purely for the evolution of resistance. Other operational factors also need to be considered. On the positive side a mixture is likely to kill more vectors and may also limit their ability to transmit the disease. On the negative side a mixture may be more expensive. Other attributes of insecticides such as their repellency are also relevant. Insecticides with some repellency will reduce exposure to the insecticide and thus, as shown here, are likely to slow the evolution of resistance and favour mixtures over sequences. However, the reduced exposure resulting from repellency also reduces the population protection provided by the insecticide [19].

This model would predict that the best strategy for limiting the development of resistance in the short term would be to use no insecticide. Of course, using no insecticide is likely to have serious consequences for mosquito and malaria abundance. The challenge is to develop good strategies for delivering mosquito and malaria control in the short term while sustaining their operational capacity in the longer term by restricting the development of resistance.

## Conclusions

This paper describes a flexible, modern model of the evolution of insecticide resistance that can help us understand the mechanisms by which different insecticide application strategies may be favoured. It replicates previous modelling work and has the potential to investigate further operational strategies for established and new insecticides. For assessing the relative benefits of mixtures and sequences it demonstrates that mixtures are likely to be favoured by high insecticide effectiveness, low exposure and high costs of resistance. Similar processes will occur in other strategies such as rotations and mosaics. This accessible modelling approach can help to promote discussion and exploration of the likely effect on resistance evolution of different insecticide use strategies in tandem with the collection of empirical data to refine and test predictions.
